# Child Welfare Exhibitions in Delhi (1920, 1924–1932): Motherhood, Public Health and Colonial Government

**DOI:** 10.1093/shm/hkae050

**Published:** 2024-12-11

**Authors:** Laura Carballido-Coria

**Affiliations:** Social Sciences Department, Universidad Autónoma Metropolitana-Cuajimalpa, Mexico City, México

**Keywords:** motherhood, public health, Delhi municipality, voluntary work

## Abstract

The child welfare exhibitions in Delhi, held for the first time in 1920, and then from 1924 to 1932, aimed at educating mothers to look properly after their children hoping to reduce illness and mortality. These exhibitions are to be understood against two broad trends. One is a worldwide interest regarding maternal and infant mortality and a greater awareness regarding the relevance of hygiene and sanitation. The other is the set of particular concerns in India and Delhi. There was a shift in policy and language between the end of the decade of 1910 and the beginning of the decade of 1920, when sanitation acquired a new meaning which included not only drainage works or cleaning of streets, but also hygiene lessons and inspection at schools; when there was talk about public health, and greater emphasis on the role of the ‘Indian public’ and ‘social service’ in the colonial discourse.

The Maternity and Infant Welfare Exhibition in Delhi, held in 1920, aimed at educating mothers to look properly after their children hoping to reduce illness and mortality. The organisation of the event involved the participation of different entities and people. On the one hand, the Delhi Deputy Commissioner, Delhi Municipality and the Association of Medical Women in India figured prominently. On the other hand, the vicereine, British and Indian male doctors, British and Indian female doctors, nurses and health visitors were there from the beginning. The Exhibition comprised lectures, exhibits, a baby show and prizes for essays on related topics. Even though the Exhibition was seen as an all-India event, the organisation was carried on by a Committee ‘which should be as representative as possible of the residents of Delhi’.^1^ Similar child welfare exhibitions were organised in the city but under the movement of the Baby Week, from 1924 till 1932.^2^ Both the Exhibition and the Baby Weeks generated much interest, according to accounts in the press and official documents, among other sources.

The Maternity and Infant Welfare Exhibition and the Baby Week are to be understood against two broad trends. One is a worldwide interest regarding maternal and infant mortality and greater awareness regarding the relevance of hygiene and sanitation. This interest was influenced by women’s movements, nationalism and imperialism and accentuated by the loss of life in conflicts such as the Franco-Prussian War, the Boer War and the First World War.^3^ Thus, official efforts translated into public health projects and regulations, but there were also voluntary organisations. In the case of Britain, for example, the Children’s Act was passed in 1908 and a National Baby Week took place in 1917; whereas in the USA a Children’s Bureau was established in 1912 and Child Welfare Exhibits were organised in 1910 in New York and in 1911 in Chicago.

The other is the set of particular concerns in India and Delhi. As Linda Bryder remarks in relation to the one held in London in 1917: ‘Baby weeks were staged elsewhere, but each must be seen as a discrete event, heavily influenced by the individuals who ran it’.^4^ There was a shift in policy and language between the end of the decade of 1910 and the beginning of the decade of 1920, when sanitation acquired a new meaning which included not only drainage works or cleaning of streets, but also hygiene lessons and inspection at schools; when there was talk about public health, and greater emphasis on the role of the ‘Indian public’ and ‘social service’ in the colonial discourse. These changes were eagerly appropriated by the Indian elite. Of course, this process was the product of an intense debate on how to make it happen. Once these changes were brought about, they helped to redefine the private and public spaces. Apart from this, the transfer of the capital from Calcutta to Delhi in 1911, demanded thinking of solutions to a city that was considered a health problem. Among the many projects devised in order to surmount this situation were the Maternity and Infant Welfare Exhibition and the Baby Week.

I am interested in analysing how these two campaigns became the site for many parallel and contending processes. No doubt, these types of activities reflected many ideas and projections about the colonised, such as the prevalence of superstitions, the zenana or child marriage. At the same time, they were an example of the quasi-governmental efforts of the colonial government, which, in this case, participated with a grant and support, while trying to enrol the support of the Indian elite and the participation of the people in general.^5^

I will begin with some remarks about relevant literature to this project, continue with a discussion about the public health policies in Delhi and a description of the origins of the Maternity and Infant Welfare Exhibition and the Baby Week, and follow it up with an analysis of the various devices meant to educate the mothers and examine the reception it received.

I have used a variety of sources: official and non-official. Official documents coming from the Deputy Commissioner Office and the Chief Commissioner Office of Delhi have allowed me to reconstruct the various projects examined here. I have also consulted several printed reports such as the Annual Report of the Public Health Commissioner. Digitised articles of *The Hindustan Times* have been particularly helpful because they followed closely the Baby weeks, both in terms of organisation and reception. I have also looked at correspondence and materials written by contemporary female doctors.

## Social Reform, Women and Motherhood

Historiography is rich in works on social reform projects, public health, maternity and childcare and women in medical careers. In his seminal book on nationalism, Partha Chatterjee has argued that Indian anticolonial nationalism was built on the idea of difference from the Western World. Chatterjee has studied the way women and the home became the centre of a series of reformist projects, which outlined what the modern Indian woman would be like in an attempt to define what Indian modernity was.^6^ However, by the end of the nineteenth-century reformist projects and associations stopped being prominent in the public agenda, since Indian nationalism demarcated a realm where only Indians could intervene: the spiritual one (the material domain pertained to the West). Dipesh Chakrabarty explored this idea too, analysing the constitution of the private and Indian modernity. One of the elements analysed by him is domestic manuals in Bengal.^7^

In their different research projects, Samita Sen, Maneesha Lal, Ranjana Saha and Mrinalini Sinha have contested Partha Chatterjee’s idea that social reform was not relevant in the twentieth century in the public agenda and that the main battles regarding womanhood took place at home. Sen agrees with Chatterjee that home became the site of resistance for the renascent nationalism that turned the education of women into an important element of the nationalist discourse: women had to be taught since they were responsible for raising the future Indian citizens. Sen states that from the 1920s onwards explanations about children’s mortality related more to the mothers’ ignorance than to poverty (poor housing and diet). According to Sen, the result of such a capitalist construction made the education of mothers more important than the framing of welfare policies.^8^

For Lal and Saha, social reform was still relevant in the twentieth century, either in the writings published in journals such as *Stri Darpan* in Northern India or in debates about infant feeding in Bengal.^9^ It is important to bear in mind that even if these projects addressed issues pertaining to ‘home’ they were carried on in the public realm. Mrinalini Sinha has argued that it is important not to reduce the fashioning of a national identity to a single moment or to lose sight of women’s participation in various movements.^10^

Ambalika Guha has explored the medicalisation of childbirth in Bengal, at the confluence of colonial politics on public health, nationalist politics and social reform. For Guha, the key element was the introduction of midwifery courses at the Calcutta Medical College.^11^

A set of texts related to women’s health care and the career of women in this area has been illuminating as to the role played by the colonial State, but also as to the participation of British and Indian women. Forbes and Burton have explored the fascination generated by the zenana among British reformers who became interested in providing medical care to Indian women. Thus, the London School of Medicine for Women, the National Association for India and the Dufferin Fund helped sustain the training of British female doctors willing to help their ‘Indian sisters’. These women found in India the justification to pursue careers that were closed to them otherwise, but despite their genuine interest in helping (and their fight against patriarchy), they also replicated hierarchies such as doctor/patient, doctor/nurse and, more importantly, viewed Indian women in the zenana as passive.^12^ Samiksha Sehrawat has written a comprehensive book on medical care in Northern India. In her chapter devoted to women and children, she analyses how the conceptions regarding the zenana moulded medical care. Her analysis of the quasi-governmental nature of many associations explores the tension between the colonisers’ commitment to civilise, to spread Western medicine, to foster associational culture, the budget constraints and the reluctance of the colonial administration to get too involved in health care. Interestingly, vicereines participated very actively in various public health projects (in their role of ‘incorporated wives’), which ended up supporting the idea of the State as separate from medical care. Sehrawat raises a very important question: what was the nature of the intervention of local, provincial and central governments in medical care as well as the impact of legislation in the way this was organised? Besides, she reflects on the history of the specialisation of medicine in India, a topic that has received little attention.^13^

In all this literature, child welfare exhibitions in Great Britain and India have received little attention, but for a few exceptions. In her long and seminal article regarding motherhood and imperialism, Anna Davin situates the origin of the Baby Week in 1917, as a result of the awareness that war implied a great loss of life, which deepened the need to educate mothers to address the problem of infant mortality.^14^ In examining this and other projects, Davin provides a very critical and insightful perspective of the way women and mothers were made responsible for infant mortality, instead of addressing the social and economic factors such as low wages.^15^ Trudi Tate elaborated on this relation between War and the Baby Week Movement. On the one hand, the great number of dead soldiers made governments wonder whether there would be enough children to protect the country and, on the other, the infant mortality made many say it was more dangerous being a baby than a soldier. For some, the origin of infant mortality lay in social conditions, but for some, it rested on ‘ignorant mothers’, for whom the different stalls and exhibitions, lectures and other activities were designed at Baby Week.^16^ For Linda Bryder, the movement initiated in Britain was strengthened by concerns about the war, but its origin predated it. Bryder sees in the movement an attempt to educate mothers, but mostly an awareness on the part of the organisers of the poverty behind infant mortality and the agency displayed by the women who were involved and who attended.^17^ Interestingly, all three authors, Davin, Tate and Bryder, point to the relevance given (or to the lack of it) to socioeconomic factors in all these projects.

In the case of the movement in India, apart from a few mentions,^18^ Siobhan Lambert-Hurley and Ranjana Saha are the ones who have written more about child welfare exhibitions. Lambert-Hurley explores the participation of the royal women of Bhopal in this and other projects (professionalisation of *dais*, promotion of sanitation and hygiene education) to provide medical care for women. There are two interesting elements in her analysis: the way these women engaged with Western medicine and adapted it to their context and the generational differences amongst them.^19^ Saha studies the Health and Child Welfare Exhibition organised in Calcutta in 1920. She uses the report of the exhibition and focuses on the lectures given during the exhibition. This allows her to explore the link established between problems in pregnancy and the construction of the ideal woman, the prejudices about the *dai* and the global nature of some ideas and concerns expressed during the Exhibition, in particular the need to regulate the baby’s pattern of feeding.^20^

My project takes these concerns about social reform, the place of women in the national question, but also the role of the various authorities in the colonial administration, as well as the changes in public health policies and furthers them in the analysis of child welfare exhibitions initiated in Delhi, in 1920.

## Delhi and Public Health

The Delhi Municipality was founded in 1863, and in the beginning, its functions had more to do with policing, levying taxes and setting up rules for life in the city.^21^ Over the years it acquired more functions, due to several changes, such as its transformation into a first-class municipality in 1871, which was accompanied by greater financial autonomy.^22^ Also, with the legal reforms introduced by Viceroy Ripon in 1881 that propounded local self-government, the Municipality acquired autonomy to use resources, along with the responsibility of getting them. This implied widening its activities and from 1887 the Municipality took charge of education.^23^

Many concerns about sanitation and disease were voiced from the beginning of the Municipality. In the period of 1858–1876, the Municipality had built a general hospital, a women’s hospital and a branch dispensary and had levied taxes to tap the Jamuna and had the city divided into *ilakas*.^24^ And in 1903, when the Durbar or ceremonial meeting to mark the proclamation of Edward VII as emperor of India was celebrated, elaborate arrangements regarding sanitation were made.^25^

Again in 1911, when another Durbar was held one of the main issues was how to protect Delhi from the plague, since the surrounding places were prone to epidemic diseases.^26^

The 1911 Durbar included a relevant development: the transfer of the British capital from Calcutta to Delhi was announced which resulted in several important decisions. The Delhi Province passed under the direct control of the government in October 1912, which implied appointing a Chief Commissioner and a Deputy Commissioner.^27^ Given the size of the old municipality of Delhi and the significance of New Delhi, several measures were taken for public health. And concerns about the site chosen were voiced in the Final Report for Town Planning.^28^

Thus, the final report on the construction of the new capital stressed that the necessary extension for the old and the new cities would have to be carefully monitored. Otherwise, the risk would be overcrowded and unsanitary places.^29^

Once the new province came about, one of the first important decisions was the appointment of a Health Officer in 1913, who was going to be in charge of improving the sanitary conditions of the cities. The need had been expressed by the Punjab government since 1908 and again in 1909, but it was only with the arrival of the colonial capital that it was finally satisfied.^30^ The reasons were not far to seek; so far there had been no need to have a health officer with high qualifications, but the foundation of New Delhi had changed things.

The Annual Report for 1913–1914 gives us an idea of the complicated tasks lying ahead not only for the Health Officer but for the Municipality too, since the statistics were not promising in terms of health. The province had serious problems of infantile and maternal mortality. To give an idea of the situation women faced (tuberculosis and problems related to maternity), the Report compared the death rate of women and men: ‘The city shows a high female death rate of 50.45 per thousand as compared with 38.78 among males’.^31^ As in the rest of India, there was no effective system of registering births and deaths. Even though several measures were devised, there remained a lot to be done. The number of patients had increased compared to the previous year, which showed the growing popularity of dispensaries and hospitals as the officer emphasised.^32^ But one can assume that the increase in patients was also due to the fact that sickness was widely prevalent.

During the decades of 1910, 1920 and 1930, the municipality of Delhi addressed the problem of sanitation and public health in myriad ways like the clearance of slums, the cleaning of the streets, the inspection of schoolchildren and the child welfare exhibitions, among others. For example, in 1915 the inspection of schoolchildren was introduced.^33^ And in 1917 two women health visitors were hired with the purpose of registering births and deaths, delivering hygiene lectures to *purdah* women and helping inspect girls in schools.^34^

However, these projects were not the result of a consensus, but were fraught with tensions about the degree of state intervention and funding, the participation of female health personnel and the dangers of interfering with the private realm.

## The Origins

At the beginning of 1919, a meeting took place in Delhi to organise a Maternity and Infant Welfare Exhibition. The idea had come up during a meeting of the Council of the Association of Medical Women in India, replicating the Exhibition held in England in 1917.^35^ Interestingly, even though in England it was called the National Baby Week, in India the name had a broader spectrum including both mothers and children.

During the next months, several meetings took place to secure a hall for the exhibition, to decide on the sections and the persons responsible for it and the lectures, magic lantern shows and exhibits. The organisation of the event gathered a series of prominent persons from Delhi: some belonging to the colonial administration, some involved in medical care and some who simply responded to the call for participation. Henry Sharp, who was in charge of the Department of Education would be the Chairman of the General Committee, whereas Col. Beadon, who was Delhi Deputy Commissioner, would be the Chairman of the Managing Committee.^36^ Dr Sethna who was the Delhi Health Officer and who designed and collaborated in several public health projects participated actively. Col. C. H. James who belonged to the Indian Medical Service was there too. Mr Ragubhir Singh was regularly involved in the following meetings. Regarding the presence of women, we have two midwifery nurses: Miss Graham and Miss Griffin, employed by the Municipality. The presence of female doctors was relevant: Ruth Young, a missionary doctor who would become a very important person in this type of project; Dr Houlton and Dr Balfour who belonged to the Women Medical Service, among others.^37^

Even though I have established the distinction between those who belonged to the colonial administration and those who did not, it is rather artificial to maintain it. This project as well as others had a quasi-governmental nature, a term used by Sehrawat to describe the Dufferin Fund and other schemes which did not have the full endorsement of the government.^38^ For Sehrawat, colonial medical care departed from the British ideal where both voluntary and state intervention were relevant: it ended up supporting medical institutions to show ‘the benevolence and paternal credentials of the colonial state’.^39^ However, the result was not clear cut, it could lead to the creation of institutions that were part of the government (such as the Central Research Institute, Kasauli), but it could also lead to the creation of movements or organisations that were not, such as the Dufferin Fund: a charity founded in 1885 by the vicereine where she and other officers’ wives could work to provide medical care for Indian women. The Fund received subscriptions of varying amounts with which it gave scholarships to women to train as doctors and nurses, funded hospitals and dispensaries and was involved in scientific research. Besides, the Fund coordinated the work of charitable institutions. Even though it aimed at preventing illness, most of its work was curative.^40^

The Exhibition was seen clearly as an initiative based on voluntary efforts but with active participation of colonial authorities and the vicereine. The Government gave a grant of Rs. 5,000, but appeals for donations were made. In July, a printed notice was circulated both to announce the Exhibition and to appeal for help in several ways: gifts of money, offers of exhibits and diagrams and help to translate into vernaculars, among others. The notice stated that Lady Chelmsford, the vicereine, was the patron of the Exhibition and that: ‘Subscriptions and other gifts or offers of help should be sent to the Honorary Secretary, Maternity and Infant Welfare Exhibition, Viceregal Lodge, Simla’.^41^

Finally, at a meeting in November, an estimate of the money received and the way it would be spent was done. Apart from the government grant, Rs. 275 of subscriptions had been received, the Association of Medical Women had given Rs. 300 each month and Lady Chelmsford had made a gift of Rs. 1,000. The money would be used to pay for the sections, the stalls, the medals and prizes, light, hire of chairs, preparation of the ground and for a secretary.^42^

To have a better understanding of the relevance of these notions of public participation, it is relevant to remember a change introduced by the 1919 Act: health became a provincial matter. As a result, emphasis was laid on the fact that the Indian public had to be aware of the importance of public health, but it had also to be responsible for it: ‘Health is now a provincial and transferred subject, and is therefore in the hands of the Indian public’.^43^

Over the following years, the introduction to the Annual Report of the Health Officer with the Indian Government, apart from presenting vital statistics, started insisting on the need for a ‘public health conscience’. Here, the narrative depicted a terrible situation in India, which was contrasted with the progress made in Great Britain due to voluntary work and awareness regarding hygiene.^44^

In this context, the Exhibition would contribute by educating ‘ignorant’ Indian women. Thus, high rates of infant and maternal mortality would diminish: low salaries and poor living conditions did not seem to be a problem, but the fact that cleanliness was absent from Indian homes. To give the reader an idea, for 1919, the death rate for children under 1 year of age was 26.8 per cent in Delhi.^45^ The Exhibition was composed of seven sections: ‘Pre-maternity, Maternity, Infant Welfare, Childhood, First Aid, Home Nursing, Domestic Hygiene and Sanitation’.^46^ In the debates leading to the preparation of materials for each one, the main topic was to educate people about the proper way to do things. Thus, when talking about spaces inhabited by Indians emphasis was laid that these had to be cleaned up, but both poor and rich houses could be good (or bad) places for childbirth and child raising. A telling example is the plan of Dr Sethna, Delhi City Health Officer, who was in charge of Domestic Hygiene and Sanitation. He thought of a very ambitious series of models to show the right and wrong ways of living, which included: ‘(a) A model of a thickly congested locality with narrow tortuous lanes and blind alleys; the houses to be of storeys varying fromone [sic] to three, a few back to back houses. Roofs of some houses meeting together and darkening the gullies underneath’.^47^ This model would contrast with ‘(b) A picture of a poor man’s wife in her cheap but decently kept house’.^48^

In the Infant Welfare Section, we find again several instances of the consequences of ignorance in the Indian public, as seen by the organisers. Models of poor and affluent houses were to be displayed and compared: a poor and congested room where the baby was to be delivered, compared with a poor room but with proper arrangements. A room in a ‘better-class house’, but dirty, with rags, and ill-ventilated compared with a room which had been properly prepared.^49^

Linda Bryder has said that Baby Week in London did help to address economic issues behind infant mortality, such as proper housing or food.^50^ But in the case of the Exhibition in Delhi, the treatment of poverty was much more complex. On the one hand, as we see from the models proposed by the organisers, there was an emphasis on the idea that hygienic surroundings were possible despite poor living conditions. This idea was reinforced in the prizes offered: several organisations offered gold or silver medals for lectures and models. One of those prizes would go to the plan for a model house: ‘for the best model plan of a house suited to an Indian family (a) whose income is Rs. 30 P.M. Or less, (b) whose income is over Rs. 30 P.M’. On the other hand, the several mentions of the differences in dwellings according to social class could detonate criticisms regarding the hard living conditions under colonialism.

As we have mentioned, several authors have explored the place that zenana occupied in colonial policies. The fact that Indian women were homogenised in colonial discourse (they were portrayed as living in seclusion) allowed many British and Indian women to pursue careers in medical care, as Burton, Forbes and Sehrawat have shown.^51^ This representation justified the creation of the London School of Medicine for Women and of the Women’s Medical Service for India (WMSI) in Britain and of the Lady Hardinge Medical College and Hospital for Women and Children in India, among other institutions.

However, the representation of the passive woman in need to be rescued coexisted with that of the woman who was in full charge of the domestic realm. In 1913 Charles Pardey Lukis, the Director General of the Indian Medical Service, had delivered a lecture at the London School of Medicine for Women and had announced the creation of the Women’s Medical Service for India (WMSI), which was going to be under the Dufferin Fund—rather than being a state service like the Indian Medical Service.^52^ Notably, the WMSI would be the main ally to solve the problems regarding health in India: the medical women were the only ones who could reach out to Indian women to educate them because they lived in *purdah*. Without Indian women’s participation, he emphasised, no real advance would be made in these matters, for they were the ones who decided in the domestic sphere.^53^ And in 1916, when Alice Pennell, a missionary doctor and sister of Cornelia Sorabji, wrote to W. M. Hailey, Chief Commissioner of Delhi and to the Viceroy to present a scheme for Women Health Visitors; she justified it in these terms:

It is the woman who regulates and orders the house; she

does the cooking, she cleans the pots with infected earth and

dirty water; it is she who decides on closing the only

window with poisonously dirty rags; if the husband or children

are ill, it is the mothers and mothers-in-law who shut every

avenue of air, and light coal-fires by the bed, and collect

the soiled linen of the house to receive any infected mater,

which they later wash in the water-course that passes

by the door and swiftly conveys its load of teeming bacilli

to neighbours near and far.^54^

Pennell added that particularly during childbirths, only mothers-in-law and mothers had a saying in the arrangements and that it was irrelevant if husbands had attended lectures, ‘for no man has a voice in the house at such times. The nurse visitors in Delhi would bear me out in this’.^55^

Then, it does not come as a surprise that seclusion was a very powerful idea around the Exhibition. A prize was announced for a talk that could be delivered in a zenana or another place, in accordance with the prevalent idea that Indian women would rather stay inside their houses.^56^ To favour women’s attendance it was decided that the exhibition would be *purdah* in the mornings, but only till 4 pm, so that men could go and it was decided too that Saturday and Sunday would not be *purdah*.^57^ This decision talks about the relevance accorded to the attendance of fathers. Besides, there was a direct appeal for ‘Help in persuading women in the zenanas to visit the Exhibition’.^58^

Finally, the Exhibition took place in February 1920 and not every section could be shown as suggested by every person in charge, sometimes for lack of space and sometimes for lack of money.^59^ But what emerged then and in the following years when other child welfare exhibitions were held, was the strong belief that through social activism awareness around public health could be created.

This emphasis on social activism was supported and encouraged by several quasi-governmental institutions and policies. For example, in 1919, while participating in the preparations for the exhibition, the vicereine Lady Chelmsford founded the Chelmsford League. The League would raise funds for the establishment of schools to train health visitors (like the Lady Reading Health School in Delhi), to do propaganda activities and to give grants-in-aid to work in places outside Governors’ provinces.^60^ The League also organised a Maternity and Child Welfare Conference in 1927, where specialists from all over India came to present papers on topics related such as antenatal work, the *dai* or propaganda work.^61^

Before concluding this section, I would like to elaborate on the response generated in official circles towards child welfare exhibitions, keeping in mind the various levels inside the colonial structure, but also the tensions and contradictions inside it. In doing this, I follow Raghav Kishore’s ideas regarding the need to look at the contradictions and ambiguities in colonial power. He refers to studies that have proposed that underdevelopment in contemporary Indian cities can be traced to the failures in the colonial era. Kishore argues that accepting this, it would mean that the municipalities’ policies were ‘consistent’.^62^

Even though the Maternity and Infant Welfare Exhibition was an initiative by the Association of Medical Women in India, the project ended up being adopted by the colonial government, mostly by the Chief Commissioner of Delhi, the Delhi Health Officer, the Municipality and the Chelmsford League at the level of British India, not without forgetting the vicereine in turn. And from 1924 till 1932, when Baby Week was organised at the beginning of the year, the inauguration included the mentioned authorities and vicereine, apart from members of the Indian elite, both from Delhi and elsewhere in India—as we will see. There was a Committee in charge of the organisation, which appealed to various bodies for grants, but with the help of colonial officers. So, for example, in 1929 the Delhi Chief Commissioner gave a grant of Rs. 326.12 annas towards the renting of shamianas and tents.^63^ He also sanctioned the sums that local bodies were going to contribute to the organisation of Baby Week.^64^

However, the perspective of the Health Officer of India about this type of event was not always the same. The Health Officer wrote Annual Reports where he analysed the main trends in health and medical care, providing statistical information and reflecting on the situation in British India. The Report echoed the need for the involvement of the Indian population in improving the health situation, which we have mentioned before. In this context, Baby Week was mentioned several times as the type of necessary endeavour. So, for instance, after reviewing the figures of infant mortality (175.56 deaths per 1,000 births, whereas in England and Wales, it was 69 per 1,000 births^65^), the Report for 1923 stressed that the only solution lied in making people conscious of the work to be done. The Report quoted a few lines from the speeches given during the Baby Week to support this: ‘and no indictment more grave than that contained in the speech of Sir Frederick Whyte at the prize giving during the same week. This is essentially a people’s problem’.^66^ Quite conveniently, the speech by the president of the Central Legislative Assembly insisted that in all countries where progress had been made, it had been made possible by private efforts, not by the state. And in the Report for 1926, while talking about the work of the voluntary associations, it dealt with the Chelmsford League and its activities of propaganda, which included: ‘baby week exhibitions which are amongst the popular and useful of the movements which have recently arisen in India’.^67^ Subsequent Annual Reports mentioned positively the great interest generated by the event and gave estimates for the number of attendants: 12,000 persons in 1928.^68^ In their pages, it was clear that the Baby Week Committee was interacting and supporting other activities since it financed maternal and child welfare activities in rural areas of Delhi Province. This positive view was shared by the Census Officer in the 1931 Report, which attributed the existence of healthier children to the Baby Weeks.^69^

However, by the end of the decade of 1920 and the beginning of the decade of 1930, the Annual Report was becoming more critical of the general situation in India. It found that the decision of making health a provincial subject, without having a central board of health was a mistake.^70^ It also pointed to the great amount of work regarding infant welfare, regretting, at the same time, that much remained to be done.^71^ Moreover, the Report for 1932 wrote critically that better ways of doing propaganda should be thought of because most Baby Weeks were simple ‘tamashas’ with no real value.^72^ Ironically, these three ideas were directed at the very foundations on which the Baby Week movement and similar projects were built on and called for greater state intervention.

These different responses to child welfare exhibitions allow us to see the colonial regime less as a consistent body and more as a set of competing perspectives and policies, in much the same way that Kishore has analysed the difficulties experienced by the Delhi municipality.^73^

## Printing, Orality and the Body

In this section, I want to explore the various means used to promote the aims of the child welfare exhibitions. Print played an important role: for the Maternity and Infant Welfare Exhibition in 1920 a book souvenir was published and prizes for essays were distributed both for this Exhibition and for the Baby weeks. There was a clear intention to promote the writing about these topics: to elicit interest and as a sort of afterthought, to reflect about the experiences generated by these exhibitions. At the same time, lectures were used too to disseminate knowledge. Visual materials were very much present in the shape of exhibits, magic lantern shows, posters and models. And the body itself played an important part.

As we have mentioned, a book souvenir was printed for the Exhibition in 1920.^74^ The contents included four speeches, six short stories and a poem. While perusing the small book of 52 pages a question arises: Who were the readers of the text? The fact that it was written in English and it was made up of speeches and literary pieces speaks of an educated and upper or middle-class public. Its pedagogical character is evident since the main objective is to enlighten about the importance of hygiene and voluntary work, the value of Western medicine, but also the need to raise awareness about harmful cultural practices such as marrying girls at an early age, lack of appreciation for female education and the zenana. Paradoxically, the texts also depicted valuable elements of Indian culture and history.

The first couple of speeches have a more official character, for they were delivered by the Chairman of the Committee and the vicereine, Lady Chelmsford. The speech by the Chairman describes Baby Week and emphasises the relevance of raising public interest in maternity and infant care. For example, while talking about the exhibits, he praises the one showing the work done by the North-West Tannery Company in Kanpur.^75^ For him, it is an example of the responsibility of employers towards their employees and should be followed by more employers. Here we have an interesting example of how the colonial state tried to foster voluntary work, but also to elicit a responsible attitude from industrialists.

In her speech, the vicereine acknowledges the participation of people in the organisation of the Baby Week, as well as the many health issues that lied its origin.

The other two speeches were delivered by the Nawab Begam of Bhopal and her daughter-in-law, Shah Bano Begam.^76^ In both texts, the nation is conjured—there is an appeal to remind the mothers that despite their religious differences, they have a common history and a common task—that of raising strong and healthy children. They share a central idea: the duty of the Indian well-to-do ladies to help their poor sisters. Remarkably, here unlike the official correspondence depicting the organisation of the exhibition, poverty does arise as an important issue: it is seen as the source or companion to ‘ignorance and superstition’, but its own origin is not explored.^77^

It is interesting to pause to reflect on their presence at the Maternity and Infant Welfare Exhibition. Why would their presence be relevant? As women belonging both to an Indian royal family and to the zenana, they were there assuming the role of educators and nation-builders. At the same time, the Nawab Begum was there because of her interest and valuable experience in this type of project in Bhopal. As Lambert-Hurley has shown, the Nawab Begum, along with other women in Bhopal, was at the centre of three projects: the professionalisation of medical personnel, the dissemination of basic knowledge regarding childbirth, first aid and proper care among mothers and the promotion of ideas regarding sanitation and hygiene.^78^

Besides, the colonial authorities considered important the participation of these rulers (as well as of the Indian elite in general), who were there to set an example and an invitation to people to collaborate. Thus, in 1930, the successor of the Nawab Begam, Nawab Hamidullah Khan, was asked by Sir John Thompson, Chief Commissioner Delhi to declare the Baby Week open. The Nawab made a call for each city and village in India to give childhood its proper status.^79^ That same year, the Maharani of Gwalior attended and delivered a speech. She praised Lady Reading for having started this movement but also talked about similar projects in Gwalior, which involved maternity homes, professionalisation of *dais* and the promotion of hygiene education and sanitation in villages. She also mentioned the participation in this sort of work of the ‘Junior Rani Sahiba’ and of ‘the wives of our sardars and ladies of rank belonging to mercantile and other communities...’.^80^

Of the short stories in the book Souvenir, three are particularly interesting because two of them outline the dangers associated with ignorance and adherence to tradition and superstition, but the other one points to old elements in Indian culture worth remembering. The first one is ‘Rupavati’ by Jogendra Singh. It tells the story of a girl who is never sent to school, for her parents think that books have no place in a woman’s life. She is married very young and even though her in-laws take care of her, her life changes when she loses two children to illness. She goes back to her parents’ house, but she dies too. The author summarises her fate and that of many other women thus: ‘This is the story of thousands of girl mothers who resign their lives at the gates of motherhood or rear only weak and emaciated children who grow into weak and incapable men, adding to the millions of Indians who live in a state of suspended animation from the cradle to the grave’.^81^

The next story is written by Cornelia Sorabji.^82^ It has two protagonists, Shanti and Shudha, two sisters; the former diligent and always looking after her sister, the latter beautiful and selfish. Shudha gets married in an incredible ceremony, but Shanti dies on the day of the wedding while trying to take a look at the wedding procession. Three years later, Shudha falls ill, because of the way they live: ‘The drains at her husband’s great Palace were ancient and unhealthy; and the Zenana cow was tuberculous -so a friend of her husband’s had said’.^83^ Initially she is looked after by a ‘quack Homeopath’ and a ‘Priest of Magic’, but after a friend intervenes, Shuddha is placed in a clean room, looked after by nurses and given a proper diet. Shuddha recovers her health believing her sister came to feed her at night, while an Aunt believes the Priest cured her, but to the reader, it is clear cleanliness and Western medicine saved her.

These two texts outline clearly the problems to be addressed if maternal and infant mortality were to be lowered: superstition and ignorance. None of these two stories make any reference to social conditions that could lead to a critique of the colonial situation. As a matter of fact, it is interesting to notice that both of them are situated in middle-class or upper-class contexts, as the mention of the ‘great Palace’ in the story by Cornelia Sorabji clearly indicates.

The third story strikes a contrast by recreating an Indian past when children were raised healthily and carefully. ‘The Story of Madhab and his Wife’, written by The Maharani Sunnity Devi of Cooch Bihar, tells us that long ago there lived a Brahmin called Madhab and his Wife, who, despite being very poor, loved their seven children and treated them with great care.^84^ When the Goddess Shashti decided to test them by sending them six more children, their attitude did not change: even if food was scarce, they still led happy lives. The message is very clear for the reader: in older days, Indian people knew how to take care of children and despite living in poverty, they could live a healthy life.

The promotion of writing was important too, so prizes were given away for essays. For example, in the Baby Week of 1930 eleven essays were sent, mostly written ‘by girls from primary standards’.^85^ Three out of them were selected for the prize.

Lectures were delivered by the specialists themselves, sometimes in English and sometimes in Indian languages, such as Urdu. The participation of doctors and nurses and other volunteers talks about the relevance given to the child welfare exhibitions. So, for the 1926 Baby Week, Dr J. R. D. Webb talked about the inspection of children in schools as it was being done in Simla. The lecture was given almost a month before the Baby week took place (early February), but it was announced as being part of the event.^86^ In 1927, Dr Meghraj Chaddha spoke in Urdu on ‘Babies Health’.^87^ In 1928, Dr Shroff, the founder of the Eye Hospital, talked about the prevention of blindness^88^; and Dr Sethna, the Medical Officer, talked about the improvement of health.^89^

Both Sinha and Banerjee-Dube have written about the importance of considering gender relations between men and women, but also between men and other men and women and other women.^90^ The child welfare exhibitions were the result of the efforts of several women: women doctors, the vicereines, health visitors, nurses and women belonging to the Indian elite. They aspired to educate other women: ‘the poor and ignorant’, as their British and Indian benefactresses coincided in calling them. But men were there from the beginning too and not only because they occupied official positions, but because they were interested in doing so. One can understand (and expect) the participation of figures such as the Delhi Chief Commissioner, and Dr Sethna, Delhi’s Health Officer, who were there because of the positions they held. But other doctors whose names came time after time over the years were not under any obligation, such as Dr Meghraj Chaddha or Dr Shroff. We can surmise their presence was owed to a sense of responsibility in the process of educating Indian women.

At the same, time, men were there to educate other men. In 1923 a speech by a doctor called A. D. Lankester was published.^91^ He delivered the speech at the National Baby Week.^92^ The audience he had in mind is composed by Indian men from the middle and upper classes, who are already in contact with Western culture. He places several challenges and questions to Indian men: to change certain cultural practices around childbirth, to spend money on childbirth, to go beyond caste limits and to allow women to enrol as nurses, to respect nurses on the street (not to ‘prey’ on them). He asks men to change the way they spend money: they spend money on weddings and dowries, but not on childbirth. So, he asks, why not set aside a sum out of the dowry to pay for these expenses? While speaking, he left no space for doubt: men had begun to question the idea that women knew better.^93^

Almost at the end of his speech, he recounts an anecdote to show that men need to put all these ideas into action at home. He says that after giving a course for *dais*, he was approached by a Pathan orderly who had had his first child. The orderly had been present while he was teaching and said:

with pride that he had listened at the door to my teaching and had

cleansed and whitewashed his house before the event and had

warned the nurse that unless she washed herself and did all that

the doctorsahib had said, she would not get any pay!^94^

Taken together, the interest shown by male doctors in giving lectures and the anecdote told by Lankester provide a richer and more complex perspective of gender relations. On the one hand, they all point towards the relevance and awareness regarding maternal and baby welfare and the willingness to act accordingly. On the other hand, particularly the lecture by Lankester is an interesting example of men educating other men by giving them concrete instructions of what do and by illustrating them with a success story.

The last example I want to examine is the Baby show. Mothers would register their babies to establish which one was healthier, a practice that took place since the Exhibition of 1920. On the chosen day, doctors would examine babies carefully: they would weigh them, look for defects (in case they found any, they were to advise the mothers on what to do) and check that the babies’ bodies and their clothes were clean. Thus, the body itself became an exhibit.

The babies were classified into categories that were not permanent and that alluded to several things, for example, to the positive colonial experience, since children who had received attention at welfare centres were put in a separate category. The categories alluded to social class, religious communities or race too. In 1925, the classification followed was:

(a) English babies, (b) Babies of educated Indian mothers, (c) Hindu babies, (d) Mahomedan babies, (e) Babies from depressed classes, (f) babies from child welfare centres.^95^

In 1928, medals were awarded according to age categories (3–6 months, 6–12 months and 12–18 months), but another element was introduced: a prize was given to the healthiest child of ‘well-to-do-parents’ and another prize for the healthiest child ‘of other classes’.^96^ And in 1932, apart from the best boy and girl prizes, distinctions were given to the best baby: of ‘well-to-do parents’, of ‘poor parents’ and of ‘intermediate class parents’.^97^

The categories are interesting for several reasons: they placed emphasis on the responsibility and knowledge on the part of Indian mothers and families and they also reflected the largest religious communities. But categories also reflected the relation of social class to health: by dividing babies from rich and poor families there was an acknowledgement that babies may be disadvantaged because of their upbringing.^98^ In this light it is interesting to reflect about the winner of one of the prizes of the Baby Show of 1925: ‘the best baby of Health centres was won by a Chamar baby, by name Champoo’.^99^

## Reception

The child welfare exhibitions in Delhi grew in importance over the following years: they were part of a larger set of institutions and projects devoted to maternal and child welfare. In 1924, Lady Reading transformed them into a national movement, with the cooperation of the Chelmsford League and the Indian Red Cross Society.^100^ Local governments and princely states (as we have mentioned) organised exhibitions called Baby Weeks: these became popular events and we have several references about them and their coordination and exchange with the Delhi Baby Week.^101^ In 1928, it was announced that a cup named Irwin Cup would be awarded to the best campaign for a Baby Week anywhere in India, attesting to the popularity and spread of the movement.^102^ Furthermore, a competition by the National Baby Council of England was established across the empire to see which Baby Week was better organised.^103^

In Delhi, *The Hindustan Times* every year from 1925 till 1932 gave exhaustive accounts of the exhibition: it reproduced the speeches by the vicereines, colonial authorities and princely rulers, it published the list of prizes given to essays, the titles of the lectures offered and sometimes even the names of the babies who won prizes for ‘Best baby’ among other details.

The newspaper also mirrored the type of reception and concerns of at least part of the population and provided a critical appraisal of the event. From the detailed articles and pictures included in some years, one can sense the interest generated by the Exhibition. Thus in 1927, the event was described in disappointing terms: it had had little assistance since tickets had been too expensive: each ticket was 4 annas, and even women had to pay that price. The Correspondent wrote that: ‘very few people, especially those who wanted to be benefited (the middle and the poor classes)’, could not attend.^104^ And in 1929, the newspaper published a brief news stating that the Secretary of the Delhi Health and Baby Week had requested more funds from the Municipality. It added that the Municipality had been giving 3,000 annually since 1926.^105^ One cannot help to notice that 9 years after the first Baby Week, there was expectation surrounding the event, but also a sense that the colonial authorities were now answerable as to the outcome of the event. This is an interesting turn in the project, particularly if we remember that the colonial authorities saw it as a way to awaken the Indian population and to educate women, but not as a responsibility to be shouldered.

By contrast, the Exhibition of 1932 was described by *The Hindustan Times* as a success:

It is impossible to estimate how much of lasting value was

absorbed by the 3000 or 4000 women, who were present

on the Purdah Day alone, but their general good behaviour

and the intelligence of the questions asked, were remarked

upon by many observers.^106^

Two elements are relevant: the high number of women attending, and the type of questions asked. They both speak of women’s interest in the topic, but the second one emphasises the knowledge they already had to be able to make questions.

That women were excited in participating is supported by another fact: the number of babies they registered for the Baby shows. In 1925 due to the amount of babies, the registration was limited to 600 hundred;^107^whereas in 1929 the number of babies registered by their mothers was over 500^108^ and in 1930 nearly 700 babies were registered.^109^

Let me give one last example of the interest generated. This came from one medicine student from Lady Hardinge Medical College, Kailash Kishori Hakser who wrote a play called ‘Dowry’. The play was presented by her classmates in one of the Baby Weeks:

she had written a Hindu play called the Dowry, which the

students acted at the Delhi Health and Baby Week,

before large audiences of women, who were deeply

moved. The dramatic movement is when the girl is

about to throw herself into the well, to avoid ruining her

family by the payment of her dowry, and a Hindu ascetic

lady appears on the scene, and call her to a life of service,

saying ‘Take the Almighty as thy husband, and the orphans

of the world as thy sons and daughters’.^110^

This brief account was written by G. J. Campbell, Principal of the institution and throws light on several elements: the appeal the Baby Week exercised in the health community and population in general in Delhi and the many ways to collaborate that surrounded the event. Besides, the two references at Indian traditions showed an interesting turn: the possibility of discarding some elements (dowry), while preserving some others (the Hindu ascetic lady).

## Final Considerations

The child welfare exhibitions held in Delhi became for a little more than 10 years not only a very popular way to carry out propaganda about health and maternal and infant welfare but also a movement that attracted the participation of many important figures in Delhi and India in general. Each one of these events was marked by the presence of the Delhi Chief Commissioner and vicereine in turn, embracing the responsibility to spread Western medicine amongst the colonised. Apart from them, prominent members of the Indian elite participated actively. While doing so, they appropriated scientific language and adopted the idea of the need of social activism to improve the health situation. Moreover, in their speeches and written communications, a sense of responsibility towards their fellow citizens emerged.

The quasi-governmental nature of the child welfare exhibitions was evident. The colonial authorities participated actively: they attended meetings to organise the event, as well as the inaugurations. They also delivered speeches, gave their support and made donations. Even though this enthusiasm aimed at awakening the conscience and responsibility of the Indian people in this and other projects related to health care. Thus, the Delhi Municipality gave an annual grant for Baby Week, but without being formally in charge of organising it, since this task was carried out by a Committee. Other organisations involved in one way or another with Baby Week had the same nature such as the Lady Hardinge College and the Chelmsford League, which were non-official, even though they could receive funds from the government and were clearly offering services that a colonial state could offer its citizens. Furthermore, the Baby Week Committee financed activities of maternal and child welfare in Delhi rural areas reinforcing and replicating the idea of voluntary work as a key element in providing health care.

It is also important to consider the unintended consequences of colonial projects as Kishore has asked us to do, which in this case was the contribution towards the consolidation of welfare state personnel.^111^ The British and Indian women who wanted to pursue a career in medical care as doctors, nurses and health visitors found a way to do it in child welfare exhibitions and related projects. Another unintended consequence of these initiatives was its contribution towards the need to conceive maternal and child care as separate areas, with specific problems and needs. For example, if we take the Annual Health Reports, we will see that in 1927 for the first time, a section devoted to this topic was included. And in 1931, it was decided to merge the Lady Chelmsford League with the Indian Red Cross Society, since both organisations oversaw childcare in India.^112^ Thus, the Indian Red Cross, another quasi-governmental institution, would have a Maternity and Child Welfare Bureau, directed by Dr Ruth Young, a specialist in the topic.^113^

The Baby Week pointed to the duality regarding the perception of mothers: on the one hand, they became answerable to the nation, for they had to raise healthy babies. The alarming maternal and infant mortality rates were caused by their ignorance, not by the economic and social conditions of the colony.

On the other hand, mothers were to be pulled out of seclusion and educated. Even if the exhibitions reinforced seclusion since there were zenana days and spaces, they were actively used by women. These ideas blurred the distinction between private and public spheres: the home was to be the site of all these policies.

Finally, to assess to what extent women embraced this type of knowledge and activities, we have various elements to consider. First, the participation as health professionals of different types, as members of the elite such as the princesses and as wives of colonial figures. Second, the high number of women who attended the exhibitions (on *purdah* and non-*purdah* days) and the number of mothers who registered their babies for Baby Shows.

## Footnotes

1 Letter from [illegible signature] to H. C. Beadon, Deputy Commissioner, Delhi, 6th (month illegible) 1919, Infant Welfare Exhibition in Delhi during 1919–1920, Deputy Commissioner Office, Delhi, DC. 12/1919, Department of Delhi Archives, henceforth DDA.

2
*Report of the Maternity and Child Welfare Conference, Held at Delhi, 4th–8th February 1927* (Delhi: Lady Chelmsford All-India League for Maternity and Child Welfare [s.d.]). In 1933 preparations had been made and a grant from the Municipal Committee was received, but due to a small-pox epidemy the event was postponed. I have not found later mentions to it. ‘Health and Baby Week’, *The Hindustan Times*, 14 January 1933, 9.

3 Anna Davin, ‘Imperialism and Motherhood’, *History Workshop*, 1978, 5, 9–65; Seth Koven and Sonya Michel, ‘Womanly Duties: Maternalist Politics and the Origins of Welfare State in France, Germany, Britain and the United States, 1880–1920’, *The American Historical Review*, 1990, 95, 1076–108; Linda Bryder, ‘Mobilising Mothers: The 1917 National Baby Week’, *Medical History*, 2019, 63, 2–23.

4 Linda Bryder, ‘Mobilising Mothers’, 4.

5 Samiksha Sehrawat uses the term quasi-governmental to characterise the projects related to medical care that the colonial state introduced, without conceiving them as state institutions, but as a combination of a commitment to modernise without assuming full responsibility for them. Samiksha Sehrawat, *Colonial Medical Care in North India: Gender, State and Society c. 1840–1920* (New Delhi: Oxford University Press, 2013).

6 Partha Chatterjee, *The Nation and Its Fragments: Colonial and Postcolonial Histories* (New Jersey: Princeton University Press, 1993).

7 Dipesh Chakrabarty, ‘La poscolonialidad y el artilugio de la Historia: ¿Quién habla en nombre de los pasados “indios”?’ in Saurabh Dube, ed, *Pasados Poscoloniales. Colección de ensayos sobre la nueva historia y etnografía de la India* (México: El Colegio de México, 1999), 639–48.

8 Samita Sen, ‘Motherhood and Mothercraft: Gender and Nationalism in Bengal’, *Gender and History*, 1993, 5, 231–43.

9 Maneesha Lal, ‘The Ignorance of Women in the House of Illness: Gender, Nationalism and Health Reform in Colonial North India’, in Bridie Andrews and Mary P. Sutphen, eds, *Medicine and Colonial Identity* (London and New York: Routledge, 2003), 14–40; Ranjana Saha, ‘Milk, Mothering and Meanings, Infant Feeding in Colonial Bengal’, *Women’s Studies International Forum*, 2017, 60, 97–110.

10 Mrinalini Sinha, ‘Refashioning Mother India: Feminism and Nationalism in Late Colonial India’, *Feminist Studies*, 2000, 26, 623–44.

11 Ambalika Guha, *Colonial Modernities: Midwifery in Bengal c 1860–1947* (London and New York: Routledge, 2018).

12 Geraldine Forbes, ‘Medical Careers and Health Care for Indian Women: Patterns of Control’, *Women’s History Review*, 1994, 3, 515–30; Geraldine Forbes, *Women in Modern India* (Cambridge: Cambridge University Press, 2004); Antoinette Burton, ‘Contesting the Zenana: The Mission to Make Lady Doctors to India 1874–1885’, *Journal of British Studies*, 1996, 35, 368–97.

13 Samiksha Sehrawat, *Colonial Medical Care in North India*.

14 Anna Davin, ‘Imperialism and Motherhood’, 43.

15
*Ibid.*, 12.

16 Trudi Tate, ‘King Baby: Infant Care Into the peace’, in Trudi Tate and Kate Kennedy, eds, *The Silent Morning: Culture and Memory Into the Armistice* (Manchester: Manchester University Press, 2013), 104–30.

17 Linda Bryder, ‘Mobilising Mothers’, 2–23.

18 Samita Sen, ‘Motherhood and Mothercraft’, 236; Geraldine Forbes, *Women in Colonial India: Essays in Politics, Medicine and Historiography* (New Delhi: Chronicle Books, 2005), 93–4; Ranjana Saha, ‘Milk, Mothering and Meanings’, 107.

19 Siobhan Lambert-Hurley, *Muslim Women, Reform and Princely Patronage: Nawab Sultan Begum of Bhopal* (London/New York: Routledge, 2007).

20 Ranjana Saha, ‘Motherhood on Display: The Child Welfare Exhibition in Colonial Calcutta, 1920’, *The Indian Economic and Social History Review*, 2021, 58, 249–77.

21 Narayani Gupta, *Delhi Between Two Empires 1803–1931: Society, Government and Urban Growth* (Delhi: Oxford University Press, 1981), 70–1; Raghav Kishore, *The (Un)governable City: Productive Failure in the Making of Colonial Delhi, 1858–1911* (Hyderabad: Orient Black Swan, 2020), 65–107.

22 Narayani Gupta, *Delhi Between Two Empires 1803–1931*, 83.

23
*Ibid*., 115.

24
*Ibid*., 88–9.

25 Official Papers Relating to the Coronation Durbar at Delhi, 1903. With the Memoranda on the Principal Administrative Arrangements made in Connection therewith (Calcutta, Office of the Superintendent of Government Printing, India, 1905), Library, National Archives of India, henceforth NAI. Bernard Cohn has studied the 1877 Durbar as part of a process to develop a ritual idiom to represent authority in colonial India. Originally, the Durbar was a ritual of the Mughal court and one of its key elements was the bond established between the ruler and his subject. The ruler gave a *kehlat* (series of cloths) and the recipient reciprocated with *peshkash* (gifts) or *nazar* (gold coins). In this way, the subject was incorporated in the body of the ruler. However, after 1858 this ritual was transformed in the search of constructing a ceremony that allowed the expressions of loyalty towards the colonisers. Three durbars were organised to mark the proclamation of an emperor or an empress: 1877, 1903 and 1911. Bernard Cohn, ‘Representación de la autoridad en la India victoriana’, in Eric Hosbawm and Terence Ranger, eds, *La invención de la tradición* (Barcelona: Crítica, 2012), 173–217.

26 Precautions for the protection of Delhi from epidemic disease, particularly plague in connection with the forthcoming Coronation Durbar. From L. C. Porter, Esq. Secretary to the Government of India, Department of Education, to the Secretary to the Government of the United Provinces, Sanitation Department and to the Secretary to the Government of the Punjab, Home (Medical and Sanitary) Department, 9 June 1911, Sanitary Plague A, Proceedings June 1911, no. 94, Department of Education, Government of India, NAI, 5.

27
*Report on the Administration of Delhi Province for the Year 1913–14* (Delhi: Superintendent Government Printing, India, 1914), 16.

28
*Final Report of the Delhi Town Planning Committee on the Town Planning of the New Imperial Capital: With Two Maps on the Scale of Four Inches to the Mile Within Fold* (Delhi: Superintendent Government Printing, India, 1913), Library, NAI, 711.405/D35, 2.

29
*Ibid*., 17–8.

30 Letter from E. R. Abbot, Esq. I.C.S., Offg. Revenue Secretary to Government, Punjab, Simla 5 July 1912, Appointment of Major Cook Young I.M.S. As Health Officer of the Delhi Municipality, File no. 22/ 1913, B, Proceedings, Education Department, Chief Commissioner, Delhi, DDA, 3–4.

31
*Ibid*., 53.

32
*Ibid*., 1.

33 From Dr K. S. Sethna, Health Officer, Delhi, to the Deputy Commissioner, Delhi Province, Delhi 24 May 1915, Orders passed regarding periodical Inspection of the Sanitary Condition of School Premises in the Delhi Province, Office of the Deputy Commissioner, Delhi, 1918, D.C. File 8/1918, DDA.

34 From E. D. Maclagan, Secretary to the Government of India, Department of Education, to W. M. Hailey, the Chief Commissioner, Delhi 12 April 1917, Scheme for Women Health Officers, File 1/1917, Education Chief Commissioner, DDA.

35 Minutes. Proceedings of a Meeting held at Nicholson Road, Delhi on the 20th March to consider the question of organising a Maternity and Infant Welfare Exhibition in Delhi in 1920, Infant Welfare Exhibition in Delhi during 1919–1920, Deputy Commissioner Office, Delhi, DC. 12/1919, DDA.

36 Letter from Lieutenant-Colonel H. C. Beadon, I.A., Deputy Commissioner, Delhi, to M. R. C. A. Barron, Chief Commissioner, Delhi, Delhi, 25 March 1919, Infant Welfare Exhibition in Delhi during 1919–1920, Deputy Commissioner Office, Delhi, DC. 12/1919, DDA.

37 Minutes. Proceedings of a Meeting held at Nicholson Road, Delhi on the 20 March to consider the question of organising a Maternity and Infant Welfare Exhibition in Delhi in 1920, *Ibid*.

38 Samiksha Sehrawat. *Colonial Medical Care in North India,* 103.

39
*Ibid*., 12.

40
*Ibid*., 101–3, 126–7; Antoinette Burton, ‘Contesting the Zenana’, 375.

41 Maternity and infant welfare exhibition. To be held in Delhi in February 1920, Infant Welfare Exhibition in Delhi during 1919–1920, Deputy Commissioner Office, Delhi, DC. 12/1919, DDA.

42 Minutes of proceedings of a meeting of the General Committee of the Maternity and Infant Welfare Exhibition held in the Hon’ble Mr Sharp’s office, Imperial Sectt., Delhi, on 11 November 1919, in *Ibid*.

43
*Annual Report of the Public Health Commissioner of India for 1922 with Appendices, vol*. *I* (Simla: Government of India Press, 1924) India Office Records, henceforth IOR, V/24/3659, 79.

44
*Annual Report of the Public Health Commissioner of India for 1923 with Appendices, vol*. *I* (Simla: Government of India Press, 1925), IOR, V/24/3659, 85.

45
*Annual Report of the Sanitary Commissioner with the Government of India for 1919 with Appendices and Returns of Sickness and Mortality among European Troops, Indian Troops, and Prisoners in India for the Year* (Calcutta: Superintendent Government Printing, 1921), IOR, V/24/3658, 58.

46
*Souvenir of the Maternity and Child Welfare Exhibition Delhi* (Calcutta: Superintendent Government Printing, 1920), British Library, henceforth BL, 7581 cc 27, 2.

47 Exhibits at the Proposed Babies Show in February 1920, Enclosure on Sanitation, Infant Welfare Exhibition in Delhi, 18–9.

48
*Ibid*., 18–9.

49 Scheme for maternity exhibition, Infant Welfare Exhibition, in *Ibid.*, 11.

50 Linda Bryder, ‘Mobilising Mothers’, 14–5.

51 Geraldine Forbes, ‘Medical Careers and Health Care for Indian Women’, 515–30; Geraldine Forbes, *Women in Modern India*; Antoinette Burton, ‘Contesting the Zenana’, 368–97; Samiksha Sehrawat, *Colonial Medical Care in North India*.

52 Sehrawat presents the WMSI as an example of a quasi-governmental institution. It was created to prove the ‘commitment’ of the colonial administration to medical care, but without assuming full responsibility. Thus, the colonial administration gave a grant to the foundation of the WMSI but put it under the control of the Dufferin Fund, because the Fund could do the work more cheaply. Samiksha Sehrawat, *Colonial Medical Care in North India*, 155, 179–80.

53 Charles Pardey Lukis, ‘The Medical Needs of India’, *The British Medical Journal*, 1913, 837–9.

54 Demi-official letter from Mrs Alice Pennell, Medical Missionary, North-West Frontier Province, to J. L. Maffey, Private Secretary to His Excellency the Viceroy, 10 September 1916, Scheme for Women Health Officers, File 1/1917, Education Chief Commissioner, DDA.

55
*Ibid*.

56 ‘Printed Notice on Prizes’, Infant Welfare Exhibition in Delhi, 118.

57 Minutes of proceedings of a meeting of the General Committee of the Maternity and Infant Welfare Exhibition held in the Hon’ble Mr Sharp’s office, Imperial Sectt., Delhi, on 11 November 1919, in *Ibid*., 96–7.

58 Maternity and Infant Welfare Exhibition. To be held in Delhi in February 1920, in *Ibid*.: 53.

59 Minutes of Proceedings of 2nd Meeting of Executive Committee of Maternity Exhibition held at Col. Beadon’s house on 1__? (brittle page) May, 1919, in *Ibid.*: 28–9.

60
*Annual Report of the Public Health Commissioner with the Government of India for 1927 with Appendices, vol. I*. (Calcutta: Government of India, Central Publication Branch, 1930), IOR, V/24/3660, 76.

61
*Report of the Maternity and Child Welfare Conference*, 7–10.

62 Raghav Kishore, *The (Un)governable City*, 66.

63 Letter from Mrs T. H. Symons, Hony. Secretary Delhi Health and Baby Week, New Delhi, 7 March 1929 to the Chief Commissioner, Delhi, Grant-in-aid to Delhi Health & Baby Week to Meet the Rent of Tents and Shamianas, File No. 6 (10) 1929, Chief Commissioner’s Office, Education, DDA, 1.

64 Contribution by local bodies to the Delhi Health and Baby Week, File No. 4 (8) Education Department 1929, DDA.

65
*Annual Report of the Public Health Commissioner of India for 1923*, 94–5.

66
*Ibid*., 95.

67
*Annual Report of the Public Health Commissioner with the Government of India for 1926: vol. I with Appendices* (Calcutta: Government of India Central Publication Branch, 1928), IOR V/24/3660, 217.

68
*Annual Report of the Public Health Commissioner with the Government of India for 1928: vol. I with Appendices* (Calcutta: Government of India Central Publication Branch, 1931), IOR V/24/3660, 281.

69 John Henry Hutton, *Census 1931: Vol. 1 Part I. Report* (Delhi: Manager of Publications, 1933), 155–6.

70 The Report for 1931 was very insistent on the need to create such a central body. *Annual Report of the Public Health Commissioner with the Government of India for 1931: vol. I with Appendices*. Delhi, Manager of Publications, 1933, IOR V/24/3661, 2–3.

71
*Annual Report of the Public Health Commissioner with the Government of India for 1933: vol. I with* Appendices (New Delhi: Manager of Publications, 1935), IOR V/24/3661, 2, 21.

72
*Annual Report of the Public Health Commissioner with the Government of India for 1932: vol. I with Appendices* (Delhi: Manager of Publications, 1934), IOR, V/24/3661, 114.

73 Kishore, *The (Un)governable City*, 65–107.

74
*Souvenir of the Maternity and Child Welfare Exhibition Delhi*, 52.

75 ‘The Chairman’s Address’, in *Ibid*., 3–4.

76 Nawab Begum of Bhopal, ‘The Duty Owed by Educated Indian Women to Their Countrywomen’ and Shah Bano Begam, ‘An Appeal to Indian Ladies’, in *Ibid*., 11–4, 45–52.

77 Shah Bano Begam, ‘An Appeal to Indian Ladies’, in *Ibid*., 47.

78 Lambert-Hurley, *Muslim Women, Reform and Princely Patronage*, 125.

79 ‘Proper Status for Childhood in India’, *The Hindustan Times*, 26 January 1930, 8.

80 ‘The Cult of Child Worship’, *The Hindustan Times*, 29 January 1930, 8.

81 Jogendra Singh, ‘Rupavati’, in *Souvenir of the Maternity and Child Welfare Exhibition Delhi*, 20.

82 Cornelia Sorabhi, ‘Shudha-Rani and the Fever Fiend’, in *Ibid*., 31–36.

83 Cornelia Sorabhi, ‘Shudha-Rani and the Fever Fiend’, in *Ibid*., 33.

84 Sunnity Devi, ‘The Story of Madhab and his Wife’, in *Ibid*., 37–42.

85 ‘11-Year Old Girl Self-Sacrifice’, *The Hindustan Times*, 30 January 1930, 8.

86 ‘Delhi Baby Week’, *The Hindustan Times*, 10 January 1926, 7.

87 ‘The Baby Week’, *The Hindustan Times*, 2 February 1926, 7.

88 ‘Dr Shroff’s Lecture’, *The Hindustan Times*, 22 January 1928, 9.

89 ‘Health Lecture’, *The Hindustan Times*, 24 January 1928 (page number illegible).

90 Mrinalini Sinha, ‘Refashioning Mother India’; Ishita Banerjee-Dube, ‘Menús modernos. Comida, familia, salud y género en Bengala colonial’, *Estudios de Asia y África*, 2015, 5, 593–620.

91 The publication of this speech points to the relevance of print in the movement of the Baby Week.

92 A. Lankester, *Responsibility of Men in Matters Relating to Maternity* (Lahore: Guran Ditta Mall Kapur, 1923), IOL.1947.a.2519.

93
*Ibid*., 1–2.

94
*Ibid*., 15.

95 ‘Delhi Baby Week’, *The Hindustan Times*, 3 January 1925, 3.

96 ‘Delhi Baby Show’, *The Hindustan Times*, 26 January 1928, 7.

97 ‘Baby Week Opened’, *The Hindustan Times*, 9 January 1932, 6.

98 I thank one of the reviewers for this idea.

99 ‘Delhi Baby Show’, *The Hindustan Times*, 17 February 1925, 3.

100 ‘National Baby Week 1924’, *British Medical Journal*, 25 October 1924, 786.

101 As we have mentioned before, Saha analyses the Health and Child Welfare Exhibition in Calcutta held in 1920. Ranjana Saha, ‘Motherhood on Display’, 249–77. Jennings states that the development of maternal care in Tanganyka followed patterns elsewhere in the empire, among them, the holding of Baby Weeks. Michael Jennings, ‘A Matter of Vital Importance. The Place of the Medical Mission in Maternal and Child Healthcare in Tanganyka, 1919–1939’, in David Hardiman, ed, *Healing Bodies, Saving Souls: Medical Missions in Asia and Africa* (Amsterdam and New York: Rodopi, 2006), 231–49.

102 ‘Maternity & Child Welfare’, *The Hindustan Times*, 28 January 1928, (page number illegible).

103 ‘National Baby Week. Imperial Competition’, *The Argus*, 17 August 1928, 6.

104 ‘The Baby Week’, *The Hindustan Times*, 2 February 1927, 7.

105 ‘Delhi Health and Baby Week’, *The Hindustan Times*, 27 November 1929, 8.

106 ‘Delhi Health and Baby Week’, *The Hindustan Times*, 14 January 1932, 6.

107 See footnote 95.

108 ‘Baby Week Ends’, *The Hindustan Times*, 31 January 1929, [n.p.].

109 See footnote 85.

110
*Lady Hardinge Medical College and Hospital for Women and Children*, New Delhi, 1929, Wellcome Collection, ann. Rep. WX 28-J 14 L15 1924–29, 18.

111 Raghav Kishore, *The (Un)governable City*. Koven and Michel have explored the link between maternalist movements and the creation of the welfare state. For them, women were the first to realise the need for welfare activities and states would come in second. Seth Koven and Sonya Michel, ‘Womanly Duties’.

112
*Annual Report of the Public Health Commissioner with the Government of India for 1929: vol. I with Appendices* (Calcutta: Government of India Central Publication Branch, 1931), IOR V/24/3660, 98.

113 Indian Red Cross Society, *Annual Report 1932*, IOR, ST1593, 14.

## Notes

Laura Carballido-Coria is a historian of colonial and postcolonial India. Her research interests include the various projects regarding public health in the Delhi and New Delhi municipalities 1911–1947, inquiry commissions and committees, particularly in colonial India; as well as the Partition of India. Her most recent publications are about the Bhore and Mudaliar Committees and the Royal Commission on Labour (1929).

